# Sustained Auditory Attention Ability Test (SAAAT) in seven-year-old children with cleft lip and palate

**DOI:** 10.1590/S1808-86942010000200009

**Published:** 2015-10-19

**Authors:** Isabel Cristina Cavalcanti Lemos, Mariza Ribeiro Feniman

**Affiliations:** Master's degree, speech therapist; Associate professor; adjunct professor

**Keywords:** attention, hearing, child, cleft palate

## Abstract

Cleft lip and palate (CLP) is a risk indicator to middle ear alterations, which may damage the development of auditory abilities such as attention that is essential to learn new skills, oral and written communication. Studies on attention process with CLP population are recent and poorly explored in the specific literature. Thus, this study aims to contribute with new subsidies in the field as it investigates the performance of children with CLP in Sustained Auditory Attention Ability Test (SAAAT).

**Material and Method:**

Comparison of SAAAT performance between children with CLP and children without it. Prospective study.

**Results:**

ANOVA was used as variance analysis model with two factors to study the variables such as group and gender. The CLP group showed an average of 2.5 units higher than the control group. This difference is between 0.7 and 4.4 with 95% certain.

**Conclusion:**

children with cleft lip and palate had poorer performance on SAAAT when compared to those without such craniofacial anomaly, considering attention reduction only.

## INTRODUCTION

Congenital cleft lip and palate (CLP) develop in the face during the embryonic and initial fetal stages. Clinically, this anomaly presents as lack of closure of the lips, palate, or both. It is known that otitis media with effusion (OME) is almost universally present in this population because of auditory tubal dysfunction. This is due to lack of palate muscle fusion, which supports the theory that middle ear hypoventilation may be a cause of OME. The palatal tensor and elevator muscles fail to effectively open the auditory tube, since they lack contralateral support because of the altered cartilaginous skeleton.^1^

OME is a special form of otitis media; it installs itself silently, and is characterized by the accumulation of serous or a glue-like mucous liquid in the middle ear (glue ear). Today, this disease is one of the most common causes of often bilateral hypoacusis in children up to age 10 years.^2^

Although hearing starts in the uterus, it is not sufficient for children to understand auditory information and to use it as a communication tool. They need to acquire analysis and interpretation abilities to process the sounds that have been detected by the peripheral auditory system.^3^

Children with hypoacusis because of OME may present hearing loss, since the auditory system may fail to correctly decode messages when peripheral changes are present; in this case, messages are distorted and incomplete. The development of hearing for auditory processing depends on innate and biological abilities together with an experience of the environment. When such abilities are altered, there may be poor academic performance, delayed language acquisition, difficulty in understanding speech, and learning impairment.

Conductive hearing loss in the first years of life may result in altered auditory processing, poor attentiveness, and the ensuing communication deficits and learning impairment.

Attentiveness is present every day, allowing subjects to select which endogenous and exogenous stimuli are important for performing tasks. Auditory attentiveness is the ability to be prepared for and to focus on a sound stimulus, at the same time being prepared to receive a different stimulus at any moment. It is essential for acquiring the acoustic and phonetic aspects of language patterns, which are essential for learning how to read and write. There are different types of auditory attentiveness; one is sustained auditory attention, which is the ability to focus attention for a time period.^4^

The Sustained Auditory Attention Ability Test (SAAAT), which is still being developed, was created to describe the auditory attentiveness in children; it provides data to verify whether any existing attention difficulty causes learning impairment or not. It may be applied to evaluate auditory attentiveness, checking whether children are able to hear auditory stimuli during prolonged time periods, and to respond only to a specific tone. It is an auditory vigilance tasks that measures the degree of attentiveness of a child, which is measured by the correct responses to specific linguistic clues; it also measures sustained attentiveness, which is measured by the ability of a child to maintain attention and concentrate on a tasks during a prolonged time period. Feniman et al.^5^ developed this test, applying it to 280 children aged from 6 to 11 years. The authors noted that younger children had more inattention errors and impulsiveness compared to older children, showing that scores correlate highly with age. They also found that throughout the group, the ability to maintain attention worsened as test task time increased. They concluded that the SAAAT might be useful in identifying auditory attentiveness difficulties.

A major cause of poor school performance in children is lack of attention. Attentiveness issues may be manifestations of several diseases, including Attention Deficit Hyperactivity Disorder (ADHD) and the Auditory Processing Disorder (APD), among others. There is, however, no consensus for establishing whether auditory attention difficulty is an associated component of APD or if it merely reflects an isolated deficit in the attentiveness process.

It is important to verify sustained auditory attention abilities in children with cleft lip and palate, because these children go through prolonged periods of sensory deprivation, which may cause changes in hearing, including attentiveness.

Cleft lip and palate is a risk indicator for middle ear alterations, which may impair the development of auditory abilities such as attentiveness, which is essential for learning new abilities, and oral and written communication. The study of attentiveness in the cleft lip and palate population is somewhat recent, and has been little investigated in the literature we reviewed. Thus, this study may yield additional data in this direction, as its purpose was to verify the performance of children with cleft lip and palate in the SAAAT, which assesses auditory attention processes.

## MATERIAL AND METHOD

The institutional review board approved this study (number 233/2005). It was carried out during 2005 and 2006.

The sample comprised 55 children of both genders, aged from 7 years to 7 years and 11 months; two groups were formed, as follows:

• G1: a control group, consisting of children without cleft lip and palate;
• G2: a study group, consisting of children with cleft lip and palate.

The inclusion criteria for G1 were: having no cleft lip and palate or any associated syndrome; no diagnosis of ADHD or use of any medication for this disorder; peripheral hearing within normal limits; being right-handed; and having no complaint and/or manifestation of APD. The inclusion criteria for G2 were: the presence of transforamen or post-foramen cleft lip and palate;^6^ no diagnosis of ADHD or use of any medication for this disorder; peripheral hearing within normal limits; being right-handed; no diagnosis of any syndrome.

G1 was formed by children from two public schools located in the same town as the specialized hospital in craniofacial anomalies from which G2 was formed. Invitations were sent to 190 children, from which 70 were interested in participating. Of these, 44 visited the hospital, from which 19 did not meet the inclusion criteria, and were excluded. Parents or caretakers of G1 children filled in a questionnaire through which we were able to note the indicators of risk for recurring otitis in childhood. The risk indicators parents or caretakers were presented with were: a history of gastroesophageal reflux within the first year of life; a history of frequent upper airway infections within the first three years of life; a history of frequent otites (three or more per year) during childhood; having undergone surgery to place ventilation tubes. Based on these answers, children were subdivided into two subgroups. The first subgroup consisted of 10 children with no risk indicators for recurring otitis during childhood; the second subgroup consisted of 15 children that had such indicators.

To form G2, our data processing center provided a list of all patients with cleft lip and palate or cleft palate only that had scheduled visits to the hospital from January to September 2006, that would be age 7 years at the time of the visit, and that would be available to participate in this study. There were 150 children scheduled, of which 30 did not show up, and 90 were excluded from the sample because of the inclusion criteria. Thus, 30 children were included in G2; six had post-foramen clefts, and 24 had incisive trans-foramen clefts.

The assessment for both group included signing a free informed consent form (parents or caretakers); a questionnaire to provide information for including or excluding children in the groups and to check auditory health and attentiveness; a test battery consisting of conventional auditory tests; and application of the SAAAT.^5^ The procedures were carried out in the same day by the same investigator.

The conventional auditory test battery consisted of: examination of the outer ear canal with a Heine otoscope, to exclude any factor that might interfere with the subsequent tests; pure tone audiometry with a Siemens model SD50 audiometer and Sennheiser HDA 200 headphones in a soundproofed room to investigate auditory thresholds at 250, 500, 1k, 2k, 3k, 4k, 6k and 8kHz; immittance testing with a Grason Stadler middle ear analyzer immittance meter, version 2, 226 Hz probe, to measure the tympanometric curve automatically (velocity = 50 decaPascals per second (daPa/s)), and ipsilateral and contralateral acoustic reflexes at 500, 1k, 2k and 4 kHz.

The test battery was carried out before the attention test, to exclude subjects with peripheral hearing loss or altered middle ear function.

Parents or caretakers of the children that participated in this study signed a free informed consent form after reading an information letter.

The SAAAT was applied in the same soundproofed room and with the same audiometry equipment. This test consists of dichotic presentation through headphones of 21 monosyllabic words, at a rate of one per second; these words are randomly rearranged and repeated to form a 100-word list that includes 20 occurrences of the word “no”. This list is presented six times without any interruptions, totaling 600 words throughout the test. There are 21 monosyllabic words: no (target word), foot, yes, flower, gol, train, sea, sun, want, bad, wool, bull, mine, salt, dad, gas, go, sky, now, dust, and one (pé, sim, flor, gol, trem, mar, sol, quer, mal, lã, boi, meu, sal, pai, gás, vou, céu, já, pó, um). The tester instructed each child verbally that they would hear a list of words and that they should raise their hand upon hearing the word “no”. As a training exercise before the first test, children were presented with a list of 50 monosyllabic words without interruptions; 10 of these were the word “no”, which were distributed randomly. The test was carried out only after each child understood the task. Answers were recorded on an answer form; an X (“X”) was placed next to each word that the child successfully identified. The binaural and dichotic test was applied in a soundproofed booth, using a CD players couples to a two-channel audiometer (SD 50) at 50 dBSL; the mean auditory air thresholds were noted for each ear.

Data were analyzed according to the SAAAT responses; two types of errors were possible: inattention error: when the child did not raise the hand in response to the target word (no) before the next word was presented; impulsiveness error: when the child raised the hand in response to a word other than the target word (no).

Adding the number of inattention errors to the number of impulsiveness errors resulted in a total number of test errors.

Vigilance was calculated by calculating the number of correct answers for the word “no” in each of the six presentations of the word list. This measure was calculated to verify decreased vigilance, that is, a decline in vigilance with time during the vigilance task. Decreased vigilance was expressed by calculating the correct number of answers for the word “no” in the first presentation, and the number of correct responses in the sixth presentation. The difference between these two numbers defines the decreased vigilance.

The statistical analysis consisted of two-factor analysis of variance (ANOVA) to verify the association between SAAAT results and the variables gender and group; post hoc comparisons were made using the Tukey correction. A statistically significant difference was defined when p≤0.05.

## RESULTS

The ANOVA model was applied to compare the two G1 subgroups; no statistically significant difference was found, as p=0.602 for the subgroup without indicators of risk, and p=0.367 for the subgroup with indicators of risk. The two subgroups were thus considered as one, and the indicators of risk were not taken into account as significant variables for data analysis.

There were no statistically significant gender differences for inattention (p=0.549), impulsiveness (p=0.746), total number of errors (p=0.539), and decreased vigilance (p=0.853) (Table 1).

**Table 1:** Number of subjects in each group according to gender.

	G1	G2
Female	13	13
Male	12	17

The SAAAT was applied to all participants in this study. Table 2 shows the results of this test for both groups.

**Table 2:** Mean, standard deviation (SD), minimum (Mín.), median (M) and maximum (Máx.) values for responses to the THAAS test in both groups.

Responses	Groups	N	Mean	DP	Mín.	M	Máx.
Inattention	1	25	14	12	0	13	39
	2	30	20	16	0	18	58
Impulsiveness	1	25	4	4	0	3	14
	2	30	5	5	1	4	19
Total no of errors	1	25	18	13	3	21	53
	2	30	25	18	2	23	77
Decreased	1	15	2	3	-3	4	8
Vigilance	2	30	4	4	0	4	14

The two-factor ANOVA model was applied to study the association between SAAAT results and the variables gender and group. Table 3 shows that there was a statistically significant association between decreased vigilance and the variable group (p=0.008). The cleft lip and palate group were, on average, 2.5 units higher than the control group; this difference is between 0.7 and 4.4 (95% confidence).

**Table 3:** Analysis of variance with two variable factors in the THAAS test.

Factor	Inattention	Impulsiveness	Total no of errors	Decreased Vigilance
Group	0,100	0,454	0,100	0,008*
Gender	0,720	0,623	0,653	0,689
Group*Gender	0,619	0,494	0,807	0,462

## DISCUSSION

Indicators of risk for recurring otitis in childhood were obtained through a questionnaire applied to parents or caretakers. Brody et al.^7^ (1999) and Stewart et al.^8^ (1999), however, have noted that parents are not able to define whether their children have OME because this is a silent condition that does not cause pain. Thus, the parents report is subjective, unreliable data, which supports bringing together the subgroups in G1, disregarding the information provided by parents or caretakers about the presence or absence of indicators of risk for recurring otitis.

We found that the performance of G2 (cleft lip and palate group) was lower compared to the control group for all types of SAAAT responses (Table 2). Children with cleft lip and palate had gone through prolonged periods of sensory deprivation because of anatomical changes in the middle ear,^9^, ^10^, ^11^, ^12^, ^13^ which may alter auditory processing^14^, ^15^, ^16^, ^17^, ^18^ and could underlie less efficient sustained attentiveness.

Attention involves voluntary and reflex processes, and stimulus-guided mechanisms that compete dynamically for control of immediate attention.^19^ A structured task in which subjects are asked to focus their attention on a specific target - such as the SAAAT - requires voluntary attention.

It is possible to observe the development of mental abilities and their correlation with the maturation of specific cognitive functions with a specific stage of neural development. The role of experience in shaping the mind and the brain.^19^ Experience is critical for the final growth steps and accurate synchronization of the brain's neural circuits.^20^ During the development of the nervous system, critical steps are crucial for normal results; at this point, neurons may compete for synaptic sites; at this point the nervous system then optimizes its neural connections.^21^ Therefore, development of attentiveness depends on visual and auditory stimuli for learning new language-related abilities.

Changes in the amplitude of the stimulus reaching the tympani may change the triggering rates of neurons,^19^ which demonstrate the close relation between perception of an auditory stimulus and the development of auditory attentiveness. Thus, it seems possible that sensory deprivation due to middle ear infection may affect the development of an individual's auditory attentiveness. Hugdahl et al.^22^ suggests that attention facilitates auditory processing.

It is common for the performance in a sustained auditory attention test to decrease towards the end of the test compared to the beginning.^5^ Table 3 shows that there was a statistically significant difference in decreased vigilance between children with cleft lip and palate and the control group (p=0.014), meaning that while controls had a 1 point decrease in vigilance, cleft lip and palate subjects had a 2.5 point decrease in vigilance. Keith^23^ demonstrated that attention declined less in children with no hearing loss and hyperactivity during the vigilance task compared to children with attention deficit and hyperactivity, showing that children with cleft lip and palate had a decrease in vigilance that was similar to that of children with attention deficit and hyperactivity.

The results of this study should be compared cautiously with other papers because several instruments for assessing attentiveness by using different modalities (auditory and visual) are described in the literature. The type of attentiveness that was assessed is not always specified, and is done by assessing the behavior of children;^24^, ^25^, ^26^, ^27^, ^28^, ^29^ other studies use individual tests to evaluate a specific type of attention.^30^, ^31^, ^32^, ^33^

Attention difficulties have been reported in subjects with reading impairment,^34^ dyslexia,^35^ aphasia,^36^ sclerosis,^37^

ADHD,^38^ APD,^39^ sleep deprivation,40 and cleft lip and palate.^32^^,^^28^

Studies relating sustained auditory attentiveness and cleft lip and palate were not found in our review; we thus tried to correlate this study with those that included subjects with a history of recurring otitis.^9^, ^10^, ^11^, ^12^, ^13^ We were aware that these studies were subject to methodological issues, including the retrospective studies that contain biases and imprecise methods for detecting OME.^39^^,^^41^ Since OME may be silent in over 50% of cases, it is hard to study; finding it requires careful medical surveillance independently from symptoms.^24^^,^^25^

In this study, the mean decreased vigilance value in the control group was 2, close to the value found by Feniman^5^ when standardizing the SAAAT (1.5). The mean decreased vigilance value was 4 in the cleft lip and palate group, which was lower compared to controls and to the mean value in Feniman's paper.^5^

Asbjornsen et al.^3^ applied a dichotic consonant-vowel test and found that the group with a history of otitis media performed worse in sustaining attention to auditory events, which was similar to our findings. Klausen et al.^33^ used a similar dichotic consonant-vowel test and concluded that OME affects the ability to persist and focus attention on auditory events.

Mahone^42^ applied a similar test to the SAAAT for assessing sustained auditory attentiveness on a sample of preschool children and found no difference in performance between children with and without otitis; this result differs from our findings. Arcia and Roberts^30^ used a test that evaluates visual and auditory sustained attentiveness and found that the number and duration of OME events is not associated with it.

There still is no consensus on the performance of children with a history of otitis during infancy relative to several areas other than attentiveness, such as language, behavior, academic and cognitive performance, and auditory processing.^26^^,^^43^, ^44^, ^45^, ^46^, ^47^, ^48^, ^49^ It should be noted, however, that children with cleft lip and palate go through far longer periods of sensory deprivation due to middle ear infection compared to children without this facial anomaly; thus, the cleft lip and palate population is different in this sense.

There were no statistically significant differences between genders in this study (Table 3); this finding concurs with those of Keith^23^ and Feniman^5^.

## CONCLUSION

Our results showed that children with cleft lip and palate perform worse on the SAAAT test compared to children without this craniofacial anomaly only in the variable decreased vigilance.
